# Voxel size and gray level normalization of CT radiomic features in lung cancer

**DOI:** 10.1038/s41598-018-28895-9

**Published:** 2018-07-12

**Authors:** Muhammad Shafiq-ul-Hassan, Kujtim Latifi, Geoffrey Zhang, Ghanim Ullah, Robert Gillies, Eduardo Moros

**Affiliations:** 1 Department of Physics, University of South Florida, Tampa, FL 33620 USA; 2 Department of Radiation Oncology, H. Lee Moffitt Cancer Center and Research Institute, Tampa, FL 33612 USA; 3 Department of Cancer Physiology, H. Lee Moffitt Cancer Center and Research Institute, Tampa, FL 33612 USA

## Abstract

Radiomic features are potential imaging biomarkers for therapy response assessment in oncology. However, the robustness of features with respect to imaging parameters is not well established. Previously identified potential imaging biomarkers were found to be intrinsically dependent on voxel size and number of gray levels (GLs) in a recent texture phantom investigation. Here, we validate the voxel size and GL in-phantom normalizations in lung tumors. Eighteen patients with non-small cell lung cancer of varying tumor volumes were analyzed. To compare with patient data, phantom scans were acquired on eight different scanners. Twenty four previously identified features were extracted from lung tumors. The Spearman rank (r_s_) and interclass correlation coefficient (ICC) were used as metrics. Eight out of 10 features showed high (r_s_ > 0.9) and low (r_s_ < 0.5) correlations with number of voxels before and after normalizations, respectively. Likewise, texture features were unstable (ICC < 0.6) and highly stable (ICC > 0.8) before and after GL normalizations, respectively. We conclude that voxel size and GL normalizations derived from a texture phantom study also apply to lung tumors. This study highlights the importance and utility of investigating the robustness of radiomic features with respect to CT imaging parameters in radiomic phantoms.

## Introduction

The extraction of quantitative information from medical images (Radiomics) holds great potential for cancer prediction and monitoring of therapy response^1–3^. These radiomics tools are promising for adding a quantitative component to existing qualitative measures and advancing personalized medicine in oncology^4^. However, there are a number of challenges that need to be addressed before implementation of any radiomic metric into the oncology workflow. These challenges include the standardization of imaging parameters and protocols, development of reliable and consistent segmentation tools, harmonization of feature extraction methods and consensus on subsequent prediction models^5,6^. Particularly, feature robustness to imaging parameters and feature extraction methods are of paramount importance to ensure successful application of CT radiomics in the field of oncology.

As recently highlighted by a number of studies^7–10^, the variability in pixel size and slice thickness in acquired CT data sets is expected if they are acquired on different scanners or using different CT protocols on the same scanner. The pixel size or reconstruction Field Of View (FOV) is an important reconstruction parameter in CT, which is not usually reported in most published radiomics papers^10^. In a lung cancer study by Basu *et al*.^11^, the variation in reconstructed slice thickness ranged from 3 to 6 mm and there was a large variability in pixel size. In another study, the pixel size ranged from 0.59 to 0.88 mm for 39 patients with metastatic renal cell cancer^12^. In a separate study, the pixel size variation was 0.39 to 0.82 mm for 33 patients, but the author resampled the volumes to isotropic voxels of length 0.59 mm using cubic spline interpolation^13^. Since both reconstructed slice thickness and pixel size determine image voxel size or number of voxels within the tumor volume (VOI), it is important to investigate feature robustness as a function of number of voxels and voxel size within VOI. The number of voxels within VOI are determined by tumor volume (VOI) and the spatial resolution of the reconstruction.

Tumor volume is a shape feature that is typically calculated in most radiomic software by multiplying voxel size by the number of voxels in the VOI. The number of voxels within the VOI, which might play a significant role in feature robustness, can be varied in two possible ways; (1) by changing the VOI while keeping the voxel size constant or (2) by changing the voxel size while keeping the VOI constant. Voxel size resampling to a selected size would be an appropriate approach to reduce or eliminate voxel size variation for most radiomic features. However, resampling is not sufficient for some intensity histogram and texture features as reported previously^8^. The important point here is that the numerical values of these features were highly correlated with number of voxels or tumor volume and this dependence can only be eliminated by including number of voxels in feature definitions (i.e., feature normalization).

The standardization of feature extraction methodology is also important for second and higher order texture features in radiomics^14^. Typically, to make feature extraction process computationally less expensive, the voxel intensities (gray levels) within the VOI are resampled to 2^N^ number of bins, where N ranges from 3 to 8 in the literature^15^. More importantly, discretization of imaging data is also used to reduce noise and increase stability of features^14,16^. Different radiomic studies have used different gray level (GL) resampling to extract features from VOI^17–19^. There could be large variability in numerical values of texture features for different discretization levels. One way to address the issue of variability due to different feature extraction techniques is to develop feature normalization methods. As recently shown, the robustness of texture features with different number of GLs significantly improves as a result of GL normalization^8^.

In this study, we validate voxel size normalizations of 10 radiomic features, derived from a texture-phantom study using 8 different CT scanners, on images of lung tumors^8^. The Spearman rank correlation coefficient (r_s_) was used to evaluate the correlation between numerical values of these radiomic features with the number of voxels before and after normalization. Moreover, 17 different texture features were extracted using different intensity discretization levels to evaluate GL normalization. The interclass correlation coefficient (ICC) was used as an assessment metric for features robustness for varying number of gray levels.

## Methods

### Patient and phantom images

This retrospective study was approved by University of South Florida (USF) institutional review board (IRB). A total of 18 patients with non-small cell lung cancer (NSCLC), with varying volumes from 4 to 123 cm^3^, were included for this study. The patients were treated with Stereotactic Body Radiation Therapy (SBRT) between 2009 and 2013. All patients’ simulation CT scans were acquired with a Brilliance Big Bore scanner (Philips Medical systems, Cleveland, OH, USA). The pixel size of the reconstructed images was 0.98 mm for two patients and 1.17 mm for rest of the patients. The reconstructed slice thickness for all patients was 3 mm. Images from four patients were reconstructed with ‘standard’ reconstruction kernel while all others were reconstructed with a ‘sharp’ kernel. One of the scans was acquired with 140 kVp and all others with 120 kVp. The range of tube current used was 65 to 483 mA.

The Credence Cartridge Radiomic (CCR) phantom^7^ scans were acquired on 8 different scanners from three major manufacturers, namely, Philips, General Electric (GE), and Siemens Healthcare systems. The scanner models were Philips Brilliance Big Bore, Philips Brilliance 64, GE Discovery STE, GE Lightspeed 32 pro, Siemens Definition AS, Siemens Sensation 64, Siemens Sensation 40, and Siemens Sensation 16^8^. The reconstructed pixel size and slice thickness were 0.98 mm and 3 mm for all phantom scans. Images were acquired using 120 kVp and 250 mA. The “standard” kernel was used for reconstruction for Philips and GE scanners while the B31f kernel was used for the 4 Siemens scanners.

### Data resampling & feature extraction

In this work, the parameter ‘number of voxels’ within a VOI was varied in two ways; (1) by changing the VOI while keeping the voxel size constant or (2) by changing the voxel size while keeping the VOI constant. In the first case, ‘number of voxels’ variation was obtained from original patient group having volumes from 4 cc to 123 cc (n = 18) with fixed voxel size. In the second case, VOI for each patient, contoured by an expert radiation oncologist, was down- and up-sampled to various voxel sizes using linear interpolation^8^. An original VOI was resampled to 4 different pixel sizes from 0.58 to 1.38 mm and 6 different slice thicknesses from 1 to 4 mm. There was therefore a total of 198 non-normalized data sets [18 patients x 11 (original + 10 resampled)]. For phantom scans, a VOI of 14.2 cc was contoured within the rubber cartridge of the CCR phantom, using an automatic contouring tool (Mirada RTx 1.6, Mirada Medical, Oxford, UK) for all scanners^8^. This VOI was again further resampled to different voxel sizes identically to the patient scans. In the case of the phantom, there was a total of 88 non-normalized data sets [8 scanners x 11 (original + 10 resampled)]. Twenty four radiomic features were extracted as follows: 4 from intensity histogram, 11 from GLCM, 4 from GLRLM, 1 from GLSZM and 4 from NGTDM. These terms and features are listed in Table 1. The first order features were calculated from the intensity based volume histograms. Second order features based on GLCM were initially developed by Haralick *et al*.^20,21^. GLCM features provide spatial dependence of gray levels of neighboring voxels as described by Oliver *et al*.^22^. The GLRLM features were implemented according to definitions provided by Galloway, Chu *et al*., and Dasarathy and Holder^23–25^. NGTDM and GLSZM based features were calculated as described by Amadasun *et al*. and Thibault *et al*. respectively^26,27^. In case of voxel size normalization, 64 equispaced gray levels were used for calculating the GLRLM, GLSZM, and NGTDM for binning the intensities of image voxels.

**Table 1:** Radiomic features analyzed in this study.

Intensity Histogram features	GLCM features	GLRLM, GLSZM & NGTDM features
1-Intensity-TGV	5-GLCM-Entropy	16-GLRLM-GLNU
2-Intensity-Energy	6-GLCM-Sum Entropy	17-GLRLM-RLNU
3-Intensity-Entropy	7-GLCM-Difference Entropy	18-GLRLM-HGRE
4-Intensity-Contrast	8-GLCM-Sum Average	19-GLRLM-SRHGE
	9-GLCM-Difference Average	20-GLSZM-HIE
	10-GLCM-Dissimilarity	21- NGTDM-Contrast
	11-GLCM-Sum Variance	22-NGTDM-Complexity
	12-GLCM-Difference Variance	23- NGTDM-Coarseness
	13-GLCM-Mean	24-NGTDM-Texture Strength
	14-GLCM-Contrast	
	15-GLCM-Inverse Variance	

Note that GLCM, GLRLM, GLSZM and NGTDM were abbreviations for gray level co-occurrence matrices, gray level run length matrices, gray level size zone matrices and neighborhood gray tone difference matrices, respectively.

### Voxel size normalization

To test the usefulness of voxel size normalization in lung cancer CT images, feature algorithms were modified by including the number of voxels, and for each feature only one of the following equations [1–3] was used.

1 $${{f}}_{{n}}(P,T)=f(P,T)\times N(P,T)$$

2 $${f}_{n}(P,T)=\frac{f(P,T)}{N(P,T)}$$

3 $${f}_{n}(P,T)=\frac{f(P,T)}{log\,[\,N(P,T)]}$$

4 $$\mathrm{where},\,N(P,T)=\frac{VOI\,}{{V}_{s}(P,T)}$$

where *f*_*n*_ *(P, T)* is the normalized feature definition, *f (P, T)* is the non-normalized feature definition as given in the pertinent cited paper, and *N (P, T)* is the number of voxels inside a VOI given pixel size ‘*P*’ and slice thickness ‘*T*’. *N (P, T)* depends both on VOI and voxel size *V*_*s*_ *(P, T)*. V_s_ (P, T) is determined both by in-plane pixel size (*P*) and slice thickness (*T*) along the longitudinal axis of the scanner. The detailed mathematical formulation used for each feature for both non-normalized and voxel size normalized cases are listed in Supplementary Table S1. In this paper, voxel size was replaced by the number of voxels inside the VOI. We note that for a given VOI, both voxel size and number of voxels within VOI provide the same information per equation 4.

### Gray level normalization

The number of GLs affects the histogram statistics and image texture. To validate the GL normalization from our phantom study^8^ in lung cancer CT images, 17 texture features including GLCM (9), GLRLM (3), GLSZM (1) and NGTDM (4) were extracted from the radiation oncologist segmented VOIs. Scan data sets were created by resampling original scans into 8, 16, 32, 64, 128 and 256 GLs for all patients/tumors. Thus, there was a total of 108 data sets (18 patients x 6 GLs) for both non-normalized and GL normalized cases. The detailed mathematical formulation for each feature for both non-normalized and GL normalized cases are listed in Supplementary Tables S2 and S3.

### Statistical analysis

The Spearman rank correlation coefficient was used as an assessment metric to evaluate the correlation between features’ numerical values and number of voxels for both non-normalized and normalized cases. The coefficient value of 1 or −1 indicates two variables are highly correlated and a value of zero indicates that there is no correlation. The absolute value of the r_s_ was calculated for 10 features to determine which features were correlated with number of voxels in the VOI before and after normalization by number of voxels. The features having values r_s_ < 0.5, 0.5 < r_s_ < 0.9, and r_s_ > 0.9 were categorized as having no, moderate, and high correlations with number of voxels respectively.

The interclass-correlation coefficient^28^ was used to evaluate the GL normalization of 17 texture features. ICC is given by equation 5,

5 $$ICC=\frac{BMS-RMS}{BMS+(d-1)\times RMS}$$

where *RMS* and *BMS* represent the between-residual and between subjects’s mean squares, and *d* is the total number of discretization levels (GL). The features having ICC < 0.5, 0.5 < ICC < 0.8, and ICC > 0.8 were categorized as not stable, intermediately stable and highly stable with respect to the varying number of GLs respectively. All statistical analysis was performed in IBM SPSS statistics version 24.0.

### Data availability

The datasets generated and analyzed during this study are available from the corresponding author on reasonable request.

## Results

### Voxel size normalization

**Figure 1:**
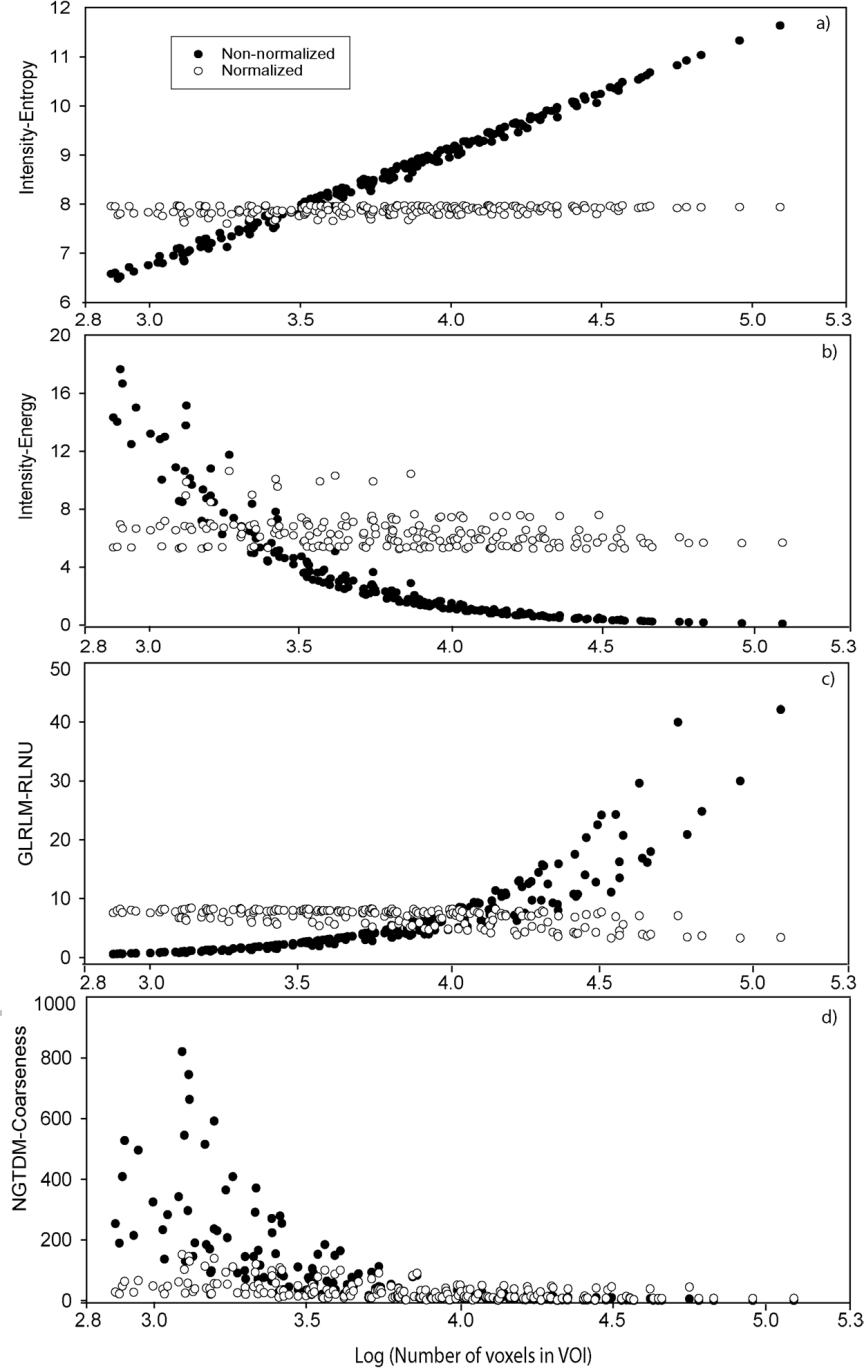
The non-normalized and normalized feature values as a function of logarithm of number of voxels for non-normalized data sets (n = 198). (**a**) Intensity-energy and (**c**) GLRLM-RLNU indicate a flat behavior, while (**b**) Intensity-energy and (**d**) NGTDM-Coarseness show small variations after normalization by number of voxels. Note that VOIs on x-axis are arranged in increasing number of voxels.

Figure 1 shows the numerical values of 4 features, extracted using non-normalized and normalized feature definitions, as a function of logarithm of the number of voxels within the VOI. VOIs were arranged according to increasing number of voxels. The non-normalized values of all four features were correlated with the number of voxels inside VOIs. However, after normalization by number of voxels, the dependence of feature values on the number of the voxels was reduced or eliminated. The intensity-entropy and GLRLM-RLNU were independent of number of voxels after normalization, mostly reflecting information about the number of voxels inside the tumor volume before normalization. In contrast, the variability of intensity-energy and NGTDM-coarseness were reduced to a lesser extent by normalization.

**Figure 2:**
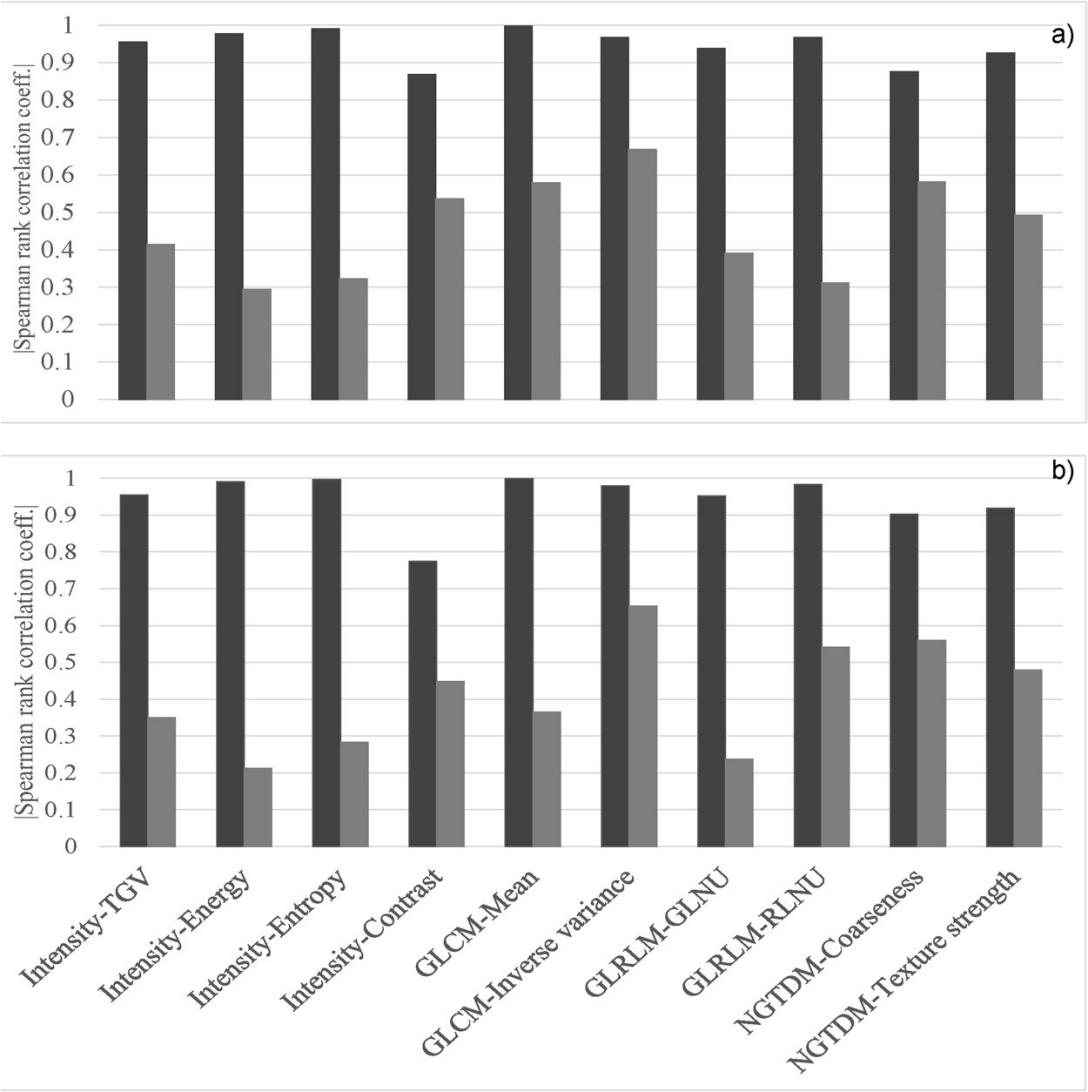
The absolute value of the Spearman correlation coefficient (r_s_) for non-normalized and normalized features for the patient cohort. (**a**) Original patient data sets (n = 18), number of voxels were varied by changing the VOI volume while keeping the voxel size constant. (**b**) Non-normalized data sets (n = 198), number of voxels were varied by down- and up- sampling the VOI of each patient to various voxel sizes. Black and gray bars represent the non-normalized and normalized features, respectively. The 95% confidence intervals for r_s_ for original (n = 18) and non-normalized data sets (n = 198) for both non-normalized and normalized feature definitions are listed in Supplementary Tables S4 and S5, respectively.

The absolute value of the Spearman rank correlation coefficient for non-normalized and normalized features for the patient cohort is shown in the Fig. 2. Figure 2a shows the coefficient value for ten features for the original 18 scan data sets. Figure 2b shows the coefficient value for the 198 non-normalized data sets which include the original and the resampled scan data sets as described in the Methods. In both cases, the value of the coefficient was between 0.9 and 1.0 for eight out of 10 original feature definitions indicating that feature values are highly correlated with the number of voxels inside the VOI. After normalization by number of voxels, most features became robust with respect to number of voxels as indicated by the low value of coefficient (r_s_ < 0.5). For most features, both the original data sets (n = 18) and non-normalized data sets (n = 198) showed similar level of correlations. Even after normalization, 4 features, namely, GLCM-inverse variance, Intensity-contrast, GLCM-mean, and NGTDM-coarseness showed moderate correlations with number of voxels (0.5 < r_s_ < 0.9) for the original scans. GLCM-inverse variance and NGTDM-coarseness were two features that showed moderate correlations with number of voxels for non-resampled data sets.

**Figure 3:**
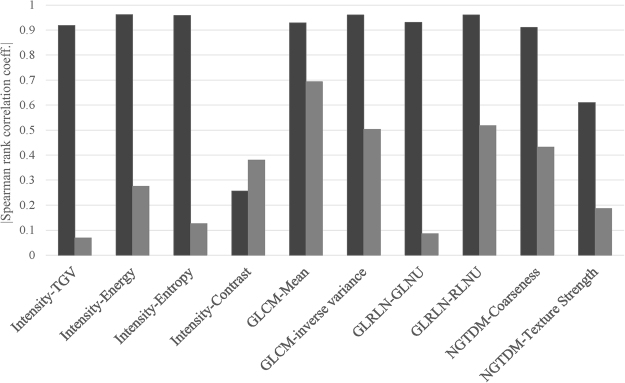
The absolute value of the Spearman correlation coefficient (r_s_) for non-normalized (black bars) and voxel size normalized (gray bars) features, extracted from the rubber cartridge of the CCR phantom (n = 88) from 8 different CT scanners. The 95% confidence intervals for r_s_ for non-normalized phantom data sets (n = 88) for both non-normalized and normalized feature definitions are listed in Supplementary Table S6.

The absolute value of Spearman correlation coefficient for non-normalized data sets (n = 88) for the rubber cartridge of the CCR phantom is shown in Fig. 3. Most features were robust with respect to number of voxels after normalization by number of voxels. The only exception was contrast based on Intensity histogram that shows no correlation with number of voxels before and after normalization (r_s_ < 0.5 for both cases).

### Gray level normalization

**Figure 4:**
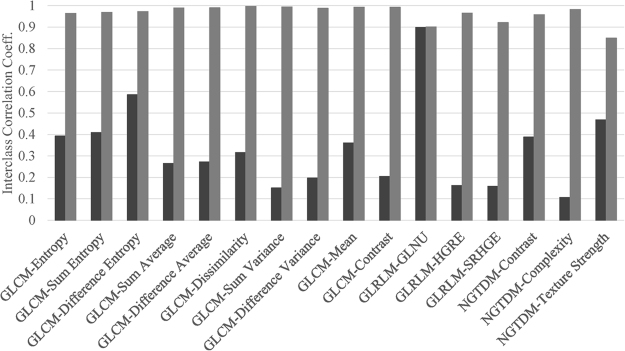
The interclass correlation coefficient (ICC) values for non-normalized (black bars) and gray level normalized (gray bars) features for lung cancer data sets (n = 108). Most features became highly stable after GL normalization (ICC > 0.8). Gray level non uniformity (GLNU) was the exception exhibiting high stability with or without GL normalization. The feature GLSZM-HIE was not plotted for clarity purposes. The 95% confidence intervals for ICCs for non-normalized data sets (n = 108) for both non-normalized and gray level normalized feature definitions are listed in Supplementary Table S7.

The ICC values for non-normalized and normalized features with varying number of GLs (n = 108) are shown in Fig. 4. Without GL normalization, most features had ICC < 0.5 indicating that features were not stable with respect to varying discretization levels. However, after GL normalization, the ICC values were between 0.8 and 1, suggesting that features became highly robust (ICC > 0.8) with respect to the number of GLs. Difference entropy derived from GLCM showed ICC value in the intermediate stability range before GL normalization. However, this feature became highly stable after GL normalization. The only exception was GLNU from GLRLM which indicated ICC close to 0.9 in both non-normalized and normalized cases. This higher value of ICC showed that this feature was independent of GL resampling. Another feature, High Intensity Emphasis (HIE) from GLSZM (not shown in Fig. 4), showed ICC value of − 0.04 and − 0.17 in non-normalized and GL normalized cases respectively. The reason for these negative values of ICC for HIE is not clear. One possibility is that the variance within the group could be greater than the variance between the groups.

## Discussion

Advanced radiomics analysis can provide useful quantitative information to supplement other clinical and –omics information thereby contributing to further development of personalized medicine in cancer treatment^4^. However, radiomics analysis for any imaging modality is affected by data acquisition and image reconstruction parameters. Therefore, one important property of a potential imaging biomarker is its robustness with respect to these parameters^10^. Most radiomic studies are currently focused on prognostic and predictive modeling while only a few reported robustness of these features with respect to imaging parameters^10^. In this study, our aim was to investigate the robustness of some CT radiomic features commonly used in lung cancer patients^29–31^ by validating our previously reported intrinsic dependencies of features using a texture phantom. We indeed showed that the voxel size and gray level normalization of CT radiomic features for lung cancer were in agreement with our previously reported findings using the CCR phantom^8^.

The importance of identifying intrinsic dependencies of radiomic features on number of voxels is exemplified by the fact that some of these features have been suggested as potential imaging biomarkers in recent studies^29,30,32–35^. For example, NGTDM-coarseness, which resembles human perception of image granularity, was found to be a useful biomarker in predicting the response to chemotherapy in NSCLC and esophageal cancer^29,32^. Coarseness was also found to be clinically useful for differentiating between normal and abnormal tissues in head-and-neck cancer patients^33^. Likewise, Intensity histogram-based energy and GLRLM-based feature grey level non-uniformity (GLNU) were suggested top performing features for predicting survival in both lung and head-and-neck cancer patients^34^. Yet in another study, GLRLM-GLNU was again suggested to have prognostic significance in adenocarcinoma^30^. Similarly, histogram-based energy was recently reported to be associated with overall survival or recurrence related survival^35^. With this in mind, it is concerning that these features were found to be intrinsically dependent on voxel size (or tumor volume or number of voxels, per equation 4) in a recent texture phantom study^8^. Therefore, this voxel size dependence raises questions regarding the reliability of these features as potential imaging biomarkers once their intrinsic dependencies are accounted for.

Resampling all CT scans to nominal voxel size is not sufficient to remove the intrinsic dependency on voxel size/VOI size/number of voxels (see equation 4) for these features. Voxel size resampling would render equal voxel size for all VOIs, but the number of voxels in each VOI will depend on tumor size per equation 4. This dependence on number of voxels is graphically explained in Supplementary Fig. S1. If CT scans were acquired with the same voxel size, normalization by number of voxels would still be required to remove the intrinsic dependence on the number of voxels, which equates to a dependence on VOI size per equation 4.

One potential way to eliminate this dependence on voxel size and VOI size is to include the number of voxels, *N (P, T)*, in mathematical definitions of these features. Note that the parameter, *N (P, T)*, depends both on VOI and individual voxel size within a VOI (equation 4). The numerical values of features were highly correlated with the number of voxels for non-normalized definitions. After normalization by number of voxels, these features became robust to both voxel size and VOI size variations (Fig. 1). This was also demonstrated by the high value of the spearman rank correlation coefficient for 8 out of 10 features for the patient cohort in Fig. 2. The coefficient value was less than 0.5 after normalization, indicating that features were not correlated with the number of voxels within the VOI (Fig. 2). Similar trend was observed for non-normalized and normalized features (Fig. 2) for varying tumor volume (n = 18) as shown in Fig. 2a and for varying voxel size (n = 198) as shown in Fig. 2b. After normalization by the number of voxels, both plots in Fig. 2a,b show similar values for the Spearman correlation coefficient in both cases. The Spearman rank correlation coefficient for original and normalized features for patients were similar to those obtained from the phantom data except for the intensity-based contrast feature shown in Fig. 3. The phantom intensity-based contrast was similar for both non-normalized and normalized features, which was contrary to our previous findings^8^. The coefficient values for Intensity-TGV, Intensity-Entropy, GLRLM-GLNU, and NGTDM-texture strength were much lower for the normalized phantom data than the patient data. This might be due to the fact that the rubber cartridge within CCR phantom contains less texture as compared to those of real lung tumors.

The dependence of some radiomics features on tumor volume has been the subject in recent studies. Fave *et al*.^36^ proposed corrected algorithms for NGTDM-Coarseness, GLRLM-GLNU, GLRLM-RLNU and Intensity-energy to remove their volume dependence which were in agreement with our results. Using the same CCR phantom^7,8^, Laure *et al*.^16^ showed that statistics energy and GLRLM-RLNU were ranked first and second in terms of their dependence on slice thickness, also in agreement with our results. However, some other features such as Intensity-entropy, GLCM-mean, GLCM-inverse variance and NGTDM-texture strength were dependent on number of voxels using both phantom scans^8^ as well as lung cancer patients scans as shown in this work. Normalization by number of voxels significantly improved these features’ robustness and therefore this normalization might be prerequisite for these features. Nonetheless, even after normalization by number of voxels, the usefulness of these features as potential biomarkers depends on many other factors^8^.

The volume dependence of identified radiomic features has implications on VOI segmentation. The robustness of radiomic features with respect to segmentation has been the topic of several recent studies^37–40^. For instance, one study reported that radiomic features were more reproducible with automatic segmentation as compared to manual segmentation^40^. It is clear that different segmentation methods may render different VOI sizes, therefore, the numerical values of identified features would also be different because of the segmentation dependent variations in VOI size. This dependence would be particularly important when comparing results across studies/institutions that used different segmentation methods.

The variability in numerical values of features due to variable gray level resampling is a challenging problem in radiomics analysis. We proposed normalization by the number of gray levels for 17 features based on our CCR phantom study^8^, and in this work we have successfully tested these definitions on lung cancer patients. Most texture features became robust to varying gray levels after gray level normalization as reflected by the higher values of the ICC (Fig. 4). Again these results are in agreement with coefficient of variation values reported in our previous paper^8^. The only exception was GLNU that showed robustness in both cases, before and after gray level normalization, contrary to coefficient of variation values in our previous report^8^. Lu *et al*.^41^ reported that three features based on GLCM including Entropy, Sum entropy and Difference entropy were robust (i.e., ICC close to 1) with varying discretization levels, contrary to our results. In our case, ICC values for these three features were less than 0.6 before normalization and close to 1 after normalization. It is possible that feature definitions employed in^41^ differ from our definitions^42^. This points to the importance of testing algorithms using virtual phantoms^4^.

## Conclusions

Previously identified clinically useful CT features such as NGTDM-Coarseness, NGTDM-Texture Strength, GLRLM-GLNU, GLRLM-RLNU, Intensity-Energy, and Intensity-Entropy depend on VOI size and voxel size. This dependence was clearly shown in this work for lung cancer patients for two different cases of varying the VOI size and the voxel size. Therefore, the previously determined voxel size normalization factors using a phantom also apply to lung cancer. Moreover, the presented gray level normalization results for texture features in this work were in agreement with the previous in-phantom results [8], except for GLRLM-GLNU that showed robustness before and after GL normalization. Therefore, we conclude that radiomics researchers should evaluate the dependence of potential imaging biomarkers to imaging acquisition parameters and gray level resampling.

## Electronic supplementary material

Supplementary Information
