# Supplementary material for: Voxel size and gray level normalization of CT radiomic features in lung cancer
Source: Sci Rep. 2018 Jul 12;8:10545. doi: 10.1038/s41598-018-28895-9 (PMC6043486; doi:10.1038/s41598-018-28895-9)
Supplement: Supplementary file 1 — Supplementary Information [file 41598_2018_28895_MOESM1_ESM.pdf]

Voxel size and gray level normalization of CT radiomic features in lung cancer: Supplementary information

M. Shafiq ul Hassan*[1,2], Kujtim Latifi[1,2], Geoffrey Zhang[1,2] , Ghanim Ullah[1], Robert J. Gillies[3] and Eduardo G. Moros[1,2]

[1]Department of Physics, University of South Florida, Tampa, FL, 33620

[2]Department of Radiation Oncology, H. Lee Moffitt Cancer Center and Research Institute, Tampa, FL, 33612.

[3]Department of Cancer Imaging and Metabolism, H. Lee Moffitt Cancer Center and Research Institute, Tampa, FL, 33612.

Corresponding Author: M. Shafiq ul Hassan, shafiqulhass@mail.usf.edu

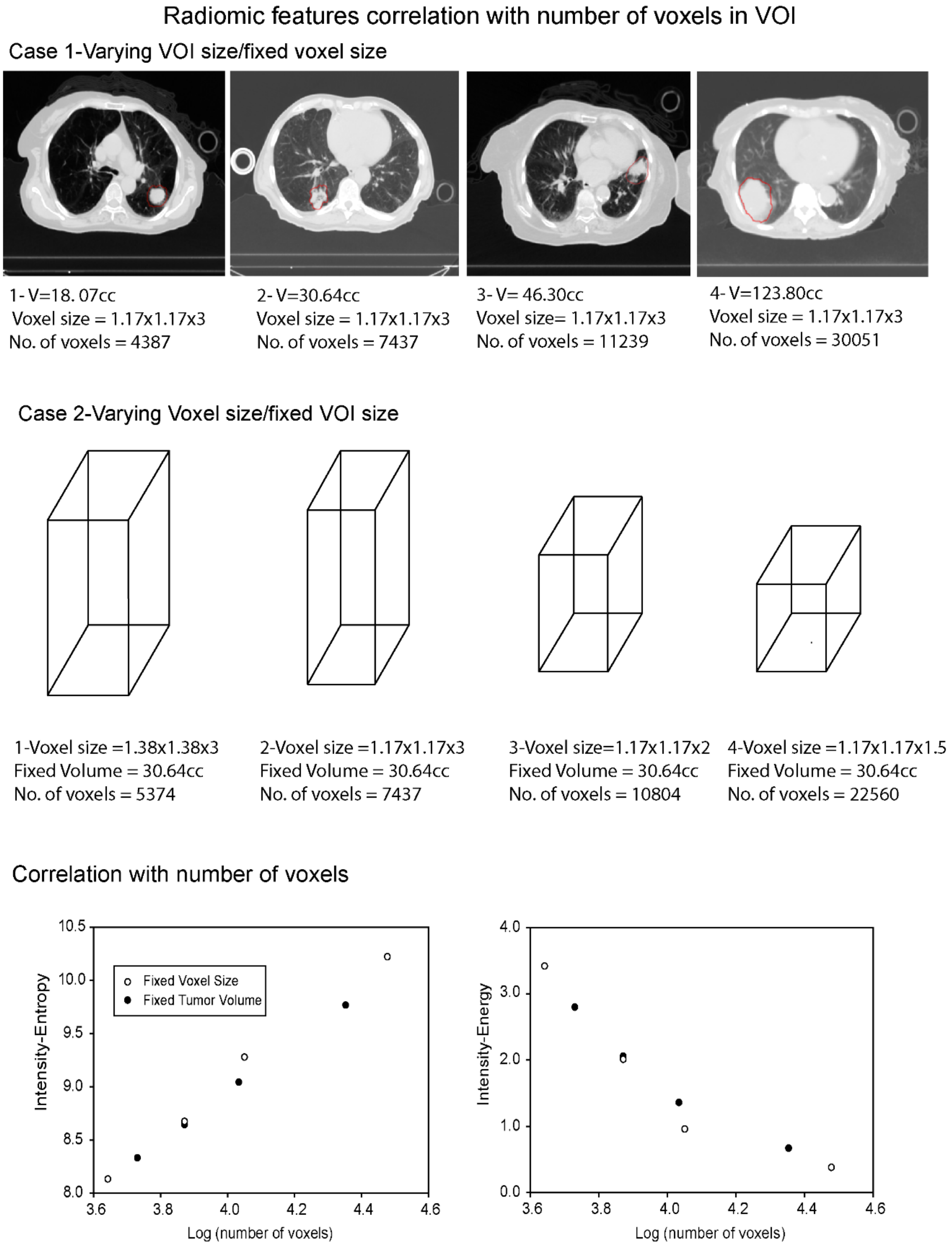

Supplementary Figure S1: Conceptual representation of radiomic feature correlation with number of voxels in VOI. In both cases, Intensity based Entropy and Energy are both highly correlated with the number of voxels whether the number of voxels is changed by varying tumor volume (VOI) with fixed voxel size or varying voxel size with fixed tumor volume.

Supplementary Table S1: Ten radiomics features from different feature groups normalized by number of voxels *N (P, T).*

Feature	Description	Non-normalized Feature formula f (P,T)	Normalized Feature formula f_n (P,T)
**First order features based on Intensity Histogram**			
1-Energy	Measures homogeneity of intensity histogram	$\sum_{x=1}^{X}\sum_{y=1}^{Y}\sum_{z=1}[T(x,y,z)]^2$	$N(P,T)*f(P,T)$
2-Entropy	Measure of disorder	$-\sum_{i=1}^{N}T(i)\log_2 T(i)$	$\frac{1}{\log[N(P,T)]}*f(P,T)$
3-TGV	Total summed intensity in ROI	$\sum_{i=1}^{G}I(v)$	$\frac{1}{N(P,T)}*f\ (P,T)$
4-Contrast	Intensity variation of intensity histogram	$\sum_{x=1}^{X}\sum_{y=1}^{Y}\sum_{z=1}^{Z}T(x,y,z)$	$\frac{1}{N(P,T)}*f\ (P,T)$
**Second order features based on Co-occurrence matrix**			
5-Inverse Variance	Place low weight on values differing from average matrix value	$\sum_{i=1}^{N}\sum_{j=1}^{N}\frac{p(i,j)}{\lvert i-j\rvert^2}, i\#j$	$\frac{1}{N(P,T)}*f\ (P,T)$
6-Mean	The mean value of the co-occurrence matrix	$\sum_{i=1}^{N}\sum_{j=1}^{N}p(i,j)$	$\frac{1}{N(P,T)}*f\ (P,T)$
**Grey level run length matrix (RLM) features**			
7-GLNU	Measures the non-uniformity of the grey levels	$\frac{1}{n}\sum_{i=1}^{M}\left[\sum_{j=1}^{N}R(i,j)\right]^2$	$\frac{1}{N(P,T)}*f\ (P,T)$
8-RLNU	Measure the non-uniformity of the run lengths	$\frac{1}{n}\sum_{i=1}^{N}\left[\sum_{j=1}^{M}R(i,j)\right]^2$	$\frac{1}{N(P,T)}*f\ (P,T)$
**Grey level Neighborhood Difference Matrix (NGTDM)**			
9-Coarseness	Measure of texture uniformity	$\left(\mathcal{E}+\sum_{i=0}^{Nh}p_i\,M(i)\right)^{-1}$	$N(P,T)*f(P,T)$
10-Texture strength	Measure of distinguishability between clusters of different intensities.	$\frac{\left[\sum_{i=0}^{N_h}\sum_{j=0}^{N_h}(p_i\ +p_j)\,(i-j)^2\right]}{\left[\mathcal{E}+\sum_{i=0}^{N_h}M(i)\right]}$ $p_i \neq 0,\ p_j \neq 0$	$N(P,T)*f(P,T)$

N (P, T) is described in text. **Histogram**: T (x, y, z) is the normalized value obtained from each voxel. T (i) is the probability of the occurrence of the grey-level *i* and N is the number of discrete intensity levels. I (v) is the intensity of a voxel, G is the number of voxels in a volume-of-interest (VOI). **GLRLM**: R (i, j) is the (i, j) th entry in the given run-length matrix and N is the number of discrete gray levels in the image. M is the longest run and n is the number of runs in the image. **NGTDM**: P_i is the probability of occurrence of voxel of intensity i and M (i) is the NGTDM value of intensity i. N_h is the highest gray level value and N is the number of grey levels present in the image.

Supplementary Table S2**:** GLCM features normalized by number of gray levels.

Feature	Non-Normalized Feature formula	Normalized Feature formula
1- Entropy	$-\sum_{i=1}^{N}\sum_{j=1}^{N} p(i,j)\, log_2\, p(i,j)$	$\frac{1}{\log[N*N]}$ * f
2- Difference Entropy	$-\sum_{i=0}^{N-1} P_{x-y}\,(i)\, log_2\{p_{x-y}(i)\}$	$\frac{1}{\log[N*N]}$ * f
3- Sum Entropy	$-\sum_{i=2}^{2N} P_{x+y}\,(i)\, log_2\{p_{x+y}(i)\}$	$\frac{1}{\log[N*N]}$ * f
4- Contrast	$\sum_{n=0}^{N-1} n^2 \left\{\sum_{i=1}^{N}\sum_{i=1}^{N} p(i,j)\right\}, \|i-j\| = n$	$\frac{f}{N*N}$
5- Mean	$\sum_{i=1}^{N}\sum_{j=1}^{N} p(i,j)$	f * N* N
6- Sum Variance	$\sum_{i=2}^{2N}(i + \sum_{i}^{N}\sum_{J}^{N} p(i,j)\log(p(i,j))^2 \log\{p_{x+y}(i)\}$	$\frac{f}{N*N}$
7- Difference Variance	$\sum_{i=2}^{2N}(i + \sum_{i}^{N}\sum_{J}^{N} p(i,j)\log(p(i,j))^2 \log\{p_{x-y}(i)\}$	$\frac{f}{N*N}$
8- Sum Average	$\sum_{i=2}^{2N} ip_{x+y}(i)$	$\frac{f}{N}$
9- Difference Average	$\sum_{i=2}^{2N} ip_{x-y}(i)$	$\frac{f}{N}$
10- Dissimilarity	$\sum_{i=1}^{N}\sum_{J=1}^{N} \|i-j\|\; p(i,j)$	$\frac{f}{N}$

p (i, j) is the co-occurrence matrix. N is the number of discrete gray levels. p_x is the ith entry obtained by summing the rows of p (i, j), p_y is the jth entry obtained by summing the columns of p(i,j).

Supplementary Table S3: GLRLM, GLSZM and NGTDM features normalized by the number of gray levels.

Feature	Non- Normalized Feature formula	Normalized Feature formula
**Gray level run length matrix (GLRLM) features**		
1- GLNU	$\frac{1}{n}\sum_{i=1}^{M}\left[\sum_{j=1}^{N} R(i,j)\right]^2$	f * N
2- HGRE	$\frac{1}{n}\sum_{i=1}^{M}\sum_{j=1}^{N} R(i,j)i^2$	$\frac{f}{N*N}$
3- SRHGE	$\frac{1}{n}\sum_{i=1}^{M}\sum_{J=1}^{N} \frac{R(i,j)i^2}{j^2}$	$\frac{f}{N*N}$
**Neighborhood gray tone difference matrix (NGTDM) features**		
4- Contrast	$[\frac{1}{N(N-1)}\sum_{i=0}^{N_h}\sum_{j=o}^{N_h} p_i\, p_j (i-j)^2][\frac{1}{n^2}\sum_{i=0}^{N_h} M(i)]$	$\frac{f}{N}$
5- Complexity	$\sum_{i=0}^{N_h}\sum_{j=0}^{N_h}\{\lvert i-j\rvert\}/(n^2(p_i + p_j))\}\{p_i\, M(i) + p_j\, M(j)\}$	$\frac{f}{N^3}$
6- Texture strength	$\frac{\left[\sum_{i=0}^{N_h}(p_i + p_j)\,(i-j)^2\right]}{\left[\varepsilon + \sum_{i=0}^{N_h} M(i)\right]}$ $p_i \neq 0,\ p_j \neq 0$	$\frac{f}{N*N}$
**Gray level size zone matrix (GLSZM) feature**		
7- HIE	$\frac{1}{\Omega}\sum_{i=1}^{m}\sum_{j=1}^{N} i^2, z(i,j)$	$\frac{f}{N*N}$

a- GLRLM: R (i, j) is the (i, j) th entry in the given run-length matrix and N is the number of discrete gray levels in the image. M is the longest run and n is the total number of runs.

b- NGTDM: P_i is the probability of occurrence of voxel of intensity i and M (i) is the NGTDM value of intensity i. N_h is the highest gray level value and N is the number of grey levels present in the image.

c- GLSZM: In size zone matrix z (i, j) rows i indicate grey levels and columns indicating zone sizes. N is the number of grey levels and the largest zone size is indicated by m. Ω is the total number of unique connected zones.

Supplementary Table S4: Spearman rank correlation coefficient (r_s) and 95% confidence intervals for original patient data sets (n = 18).

	Non-Norm. Feature Definition	95% Confidence Intervals		Norm. Feature Definition	95% Confidence Intervals	
Features	r_s	Lower	Upper	r_s	Lower	Upper
Intensity-TGV	0.955	0.881	0.983	0.414	-0.065	0.738
Intensity-Energy	-0.977	-0.991	-0.938	-0.294	-0.669	0.200
Intensity-Entropy	0.990	0.973	0.996	0.323	-0.169	0.686
Intensity-Contrast	0.868	0.675	0.949	-0.536	-0.802	-0.093
GLCM-Mean	0.998	0.995	1.000	0.579	0.154	0.823
GLCM-Inverse variance	0.967	0.912	0.987	0.668	0.293	0.865
GLRLM-GLNU	0.938	0.839	0.977	0.391	-0.092	0.725
GLRLM-RLNU	0.967	0.912	0.987	-0.311	-0.679	0.182
NGTDM- Coarseness	-0.876	-0.953	-0.693	-0.581	-0.824	-0.157
NGTDM-Text. Strength	-0.926	-0.972	-0.809	-0.492	-0.779	-0.033

Supplementary Table S5: Spearman rank correlation coefficient (r_s) and 95% confidence intervals for non-normalized data sets (n = 198).

Features	Non-Norm. Feature Definition r_s	95% Confidence Intervals Lower	Upper	Norm. Feature Definition r_s	95% Confidence Intervals Lower	Upper
Intensity-TGV	0.954	0.940	0.965	0.350	0.222	0.466
Intensity-Energy	-0.990	-0.992	-0.987	-0.212	-0.341	-0.075
Intensity-Entropy	0.997	0.997	0.997	0.282	0.149	0.405
Intensity-Contrast	0.774	0.712	0.824	-0.448	-0.552	-0.330
GLCM-Mean	0.999	1.000	1.000	0.365	0.238	0.480
GLCM-Inverse variance	0.980	0.974	0.984	0.653	0.566	0.726
GLRLM-GLNU	0.952	0.937	0.963	0.237	0.101	0.364
GLRLM-RLNU	0.983	0.978	0.987	-0.541	-0.632	-0.435
NGTDM-Coarseness	-0.902	-0.925	-0.873	-0.559	-0.647	-0.456
NGTDM-Text. Strength	-0.919	-0.938	-0.895	-0.479	-0.579	-0.364

Supplementary Table S6: Spearman rank correlation coefficient (r_s) and 95% confidence intervals for non-normalized phantom data sets (n = 88).

Features	Non-Norm. Feature Definition r_s	95% Confidence Intervals Lower	Upper	Norm. Feature Definition r_s	95% Confidence Intervals Lower	Upper
Intensity-TGV	0.918	0.878	0.945	0.069	-0.142	0.274
Intensity-Energy	-0.962	-0.974	-0.943	0.275	0.070	0.458
Intensity-Entropy	0.958	0.937	0.972	-0.126	-0.326	0.085
Intensity-Contrast	0.256	0.05	0.441	-0.380	-0.545	-0.186
GLCM-Mean	0.928	0.892	0.952	0.693	0.566	0.788
GLCM-Inverse variance	0.960	0.940	0.973	0.503	0.329	0.644
GLRLM-GLNU	0.930	0.895	0.953	0.086	-0.125	0.290
GLRLM-RLNU	0.960	0.940	0.973	-0.517	-0.655	-0.345
NGTDM-Coarseness	-0.910	-0.94	-0.866	-0.432	-0.588	-0.245
NGTDM-Text. Strength	-0.610	-0.726	-0.460	0.186	-0.024	0.380

Supplementary Table S7: Interclass Correlation Coefficient (ICC) and 95% confidence intervals for non-normalized patient data set (n = 108).

Features	Non-Norm. Feature Definition ICC	95% Confidence Intervals Lower	Upper	Norm. Feature Definition ICC	95% Confidence Intervals Lower	Upper
GLCM-Entropy	0.393	0.091	0.690	0.964	0.900	0.987
GLCM-Sum Entropy	0.409	0.096	0.704	0.969	0.931	0.988
GLCM-Diff. Entropy	0.585	0.185	0.825	0.973	0.947	0.988
GLCM-Sum Average	0.265	0.029	0.556	0.989	0.968	0.996
GLCM-Diff. Average	0.272	0.034	0.563	0.990	0.981	0.996
GLCM-Dissimilarity	0.316	0.044	0.613	0.997	0.988	0.999
GLCM-Sum Variance	0.151	-0.035	0.429	0.994	0.985	0.998
GLCM-Diff. Variance	0.197	-0.031	0.498	0.988	0.975	0.995
GLCM-Mean	0.316	0.010	0.675	0.971	0.879	0.991
GLCM-Contrast	0.205	-0.037	0.513	0.993	0.986	0.997
GLRLM-GLNU	0.901	0.807	0.958	0.899	0.755	0.961
GLRLM-HGRE	0.163	-0.038	0.451	0.965	0.892	0.987
GLRLM-SRHGE	0.158	-0.042	0.446	0.921	0.772	0.972
GLSZM-HIE	-0.004	-0.012	0.018	-0.176	-0.948	0.439
NGTDM-Contrast	0.388	0.066	0.682	0.958	0.892	0.984
NGTDM-Complexity	0.107	-0.0106	0.417	0.982	0.961	0.993
NGTDM-Text. Strength	0.849	0.710	0.935	0.468	0.106	0.748
